# Bioefficiency of microencapsulated hemp leaf phytonutrient-based extracts to enhance *in vitro* rumen fermentation and mitigate methane production

**DOI:** 10.1371/journal.pone.0312575

**Published:** 2024-10-31

**Authors:** Srisan Phupaboon, Maharach Matra, Ronnachai Prommachart, Pajaree Totakul, Metha Wanapat

**Affiliations:** 1 Tropical Feed Resources Research and Development Center (TROFREC), Department of Animal Science, Faculty of Agriculture, Khon Kaen University, Khon Kaen, Thailand; 2 Department of Animal Science, Faculty of Agriculture and Natural Resources, Rajamangala University of Technology, Tawan-Ok, Chonburi, Thailand; 3 Division of Animal Science, Faculty of Agricultural Technology, Rajamangala University of Technology Thanyaburi, Thanyaburi, Pathum Thani, Thailand; Zagazig University Faculty of Agriculture, EGYPT

## Abstract

The objective was to assess the supplementation with microencapsulation of hemp leaf extract (mHLE) utilized as a rumen enhancer on *in vitro* rumen fermentation and to enhance the bioavailability of active compounds for antimicrobial action, particularly in protozoa and methanogen populations. The feed treatments were totally randomized in the experimental design, with different levels of mHLE diet supplemented at 0, 4, 6 and 8% of total DM substrate and added to an R:C ratio of 60:40. During fermentation, gas kinetics production, nutrient degradability, ammonia nitrogen concentration, volatile fatty acid (VFA) profiles, methane production, and the microbial population were measured. The supplemented treatment at 6% of total DM substrate affected reductions in gas kinetics, cumulative gas production, and volatile fatty acid profiles, especially the acetate and acetate to propionate ratio. Whereas propionate proportion and total volatile fatty acid concentration were enhanced depending on the increase of nutrients *in vitro* dry matter degradability (IVDMD) after 12 h of post-fermentation at a R:C ratio of 60:40 (*P* < 0.05). Consequently, mHLE addition resulted in optimal ruminal pH and increased nutrient degradability, followed by ammonia nitrogen concentrations (*P* < 0.05), which were enhanced by dominant cellulolytic bacteria, particularly *Ruminococcus albus* and *Ruminococcus flavefaciens*, which showed the highest growth rates in the rumen ecology. Therefore, mHLE, a rich phytonutrient feed additive, affected the methanogen population, reduced the calculated methane production and can be a potential supplement in the ruminant diet.

## Introduction

At present, there is interest in an extensive selection of agro-nutrient plants as potential feed additives for reducing the chain of food production’s effects on the environment. Hemp (*Cannabis sativa* L.) derivatives are among the agro-nutrient products used in animal nutrition to reduce feed costs and increase the sustainability and quality of animal products, particularly meat and milk [1]. There are suggestions that hemp plant-derived products from agricultural operations can improve meat and/or milk production, shelf life, and animal health [1]. One such example is the rapidly growing hemp sector, which generates seeds, leaves, seed oil, and cake. These hemp by-products can be a valuable source of protein in the diets of ruminants, potentially replacing protein source such as soybean meal [1]. According to several studies on the nutritional value, the crude protein (CP) content obtained from hemp by-products exceeds the recommended dietary needs for ruminant growth (120–180 g/kg CP DM) and maintenance (60–110 g/kg CP DM) [2, 3]. In addition, they provide an essential amino acid profile that is well-balanced and equivalent to soybean meal, particularly in regard to methionine (1.8 and 2.0% CP) and lysine (6.4 and 6.8% CP) as per the body requirements for goats and cattle, respectively [4–6].

Semwogerere et al. [6] have discussed the bioavailability and bioefficacy of hemp phytochemicals for enhancing ruminant health, production, and increasing meat shelf life. Bioavailability of dominant bioactive compounds in hemp by-products, such as terpenoids, alkaloids, flavonoids, terpenes, phenolics, lignans, plant steroids, curcumins, saponins, glucosides, cannabinoids, and polyphenols, is added to the diet of ruminant animals to improve rumen manipulation and decrease methane production during fermentation [7], while tetrahydrocannabinol (THC) and cannabidiol (CBD) are unpublished modes of action in feed additives to mitigate methane. The most abundant secondary phytochemical constituents are phenolics, which account for 45% of all secondary phytochemical constituents in plants, followed by terpenoids and steroids (27%), alkaloids (18%), and others (10%). They have anti-inflammatory, antispasmodic, anti-allergic, antioxidants, antibacterial, antifungal, chemo preventive, neuroprotective, hypotensive, and antiaging properties [6]. The nutritional quality of spent hemp biomass and its implications for animal health, emphasizing the need for further investigation into its cannabinoid content. Industrial hemp is defined as containing CBD (>1%) and THC (0.03%, and while it can yield significant amounts of CBD, the residual cannabinoids, including CBD and THC, may remain in byproducts post-extraction. Specifically, the research indicates that spent hemp biomass can contain residual cannabinoids, which may accumulate in animal tissues and potentially affect consumer exposure [8]. Recent evidence suggests that the *in vivo* experiment based on the bioactivity profile of their byproducts showed how these plant products can be used (as an additive ≤ 3% of the total DM diet) as supplements in terms of phytonutrients and phytochemicals [6]. The previous study added hemp biomass (SHB)-based residue leaves to the diet of lactating dairy cows without affecting lactation performance or overall health, despite its lower palatability. Notably, the improved nitrogen utilization efficiency and increased milk production after SHB withdrawal suggest that SHB may enhance long-term feeding efficiency in dairy cows [8]. Although the THC level in these studies is very low or minimal, it is accumulated in the liver, meat, and fat tissues and can be transferred or exposed to consumers [7, 9]. However, many studies demonstrated that the bioavailability and bioaccessibility of hemp by-products are rich in phytonutrients and varied sources of strong antioxidants and antimicrobials that are good for *in vitro* ruminant nutrition, nutrient degradability, ruminal nitrogen, and mitigate methane emissions were supplemented in the range of 3.3 to 10% of the total DM diet [10–12]. Importantly, those essential substances are unstable and readily degradable in various environmental conditions such as temperature, pH, and light; they also have antinutritive values that restrict their use and could be of low solubility [13, 14]. The strategy of nano/microencapsulation of bioactive substances was created to address these disadvantages in this research.

Microencapsulation is a technique to retain an active ingredient inside various encapsulants such as plant-based protein, carbohydrate, and protein, especially chitin or chitosan, through spray-drying and/or freeze-drying methods, which are suitable for use in feed supplementation as rumen enhancers [15–17]. Considering the aid of microencapsulation technology, numerous industrial substances can now be employed more effectively. Like other controlled-release processes, microencapsulation allows the reformulation of various food-feed systems and pharmaceutical products, enhancing their properties and providing new functions as bioactive agents in the body [14, 16]. Several authors have emphasized the benefits and significance of using microencapsulation in those industries, particularly in the food and pharmaceutical industries [18]. It is stressed that it may protect the core compound, lessen their reactivity with outside factors, slow down the rate at which the core compound transfers to the outside, control how much of the core compound is released into the environment, encourage easier handling, cover up the taste of the core compound, and dilute the core compound in the finished product when it is toxic in large quantities. Additionally, it is easier to employ some compounds that are sensitive to heat, temperature, or pH [15, 19].

Utilizing the biodegradable chitosan microencapsulation employed to inhibit methanogenesis has long been considered a promising technique to improve ruminant production. Designing feeding strategies with methane mitigation using natural biopolymer extracts, particularly chitosan, created by deacetylating chitin obtained from crab shells, offers broad-spectrum antimicrobial effects [19–21]. Recently, *in vitro* rumen fermentation studies have shown that chitosan extract may reduce ruminal protein disappearance, thereby increasing propionate concentration and decreasing methane production, leading to more efficient fermentation patterns [17]. Moreover, Lian et al. [22] demonstrated the efficacy of chitosan microcapsules in enhancing antibacterial activity against Vibrio in shrimp culture in natural seawater. Other research findings into the microencapsulation of probiotics and prebiotics in alginate-chitosan capsules resulted in viability under heat processing in shrimp feeds subjected to 70°C for 60 min and 90°C for 5 min [23].

Therefore, this study hypothesizes applying microencapsulation to retain phytochemicals and phytonutrients from hemp plant extracts to manipulate their release within capsules. Particularly focusing on polyphenolic and antioxidative compounds, for increasing *in vitro* ruminal digestibility, changing rumen fermentation, and potentially reducing methane emissions by using it as a feed supplement. This research addresses limitations in release stabilization and optimizes bioaccessibility levels in *in vitro* experiments. This research aimed to assess the impact of supplementing feed with microencapsulated hemp leaf extract (mHLE) at varying levels (0%, 4%, 6%, and 8% of total dry matter substrate) on *in vitro* rumen fertilization, nutrient degradability, and methane (CH_4_) production.

## Materials and methods

### Animal ethics

Rumen fluid inoculum collection from the rumen-fistulated Thai-crossbred dairy steers was approved by the Institutional Animal Care and Use Committee of Khon Kaen University and the Institute of Animals for Scientific Purpose Development (IAD), Thailand (record no. IACUC-KKU 110/66 and U1-10937-2566).

### Plant material and chitosan-microencapsulated of hemp extracts

Hemp (*Cannabis sativa* L.) type KKU05 leaves were collected in March 2022 from plants cultivated in the Cannabis Research Institute, Khon Kaen University, Khon Kaen, Thailand, under greenhouse-controlled management. The collection of hemp material complied with institutional, national, and international guidelines and legislation on hemp. In accordance with guidelines and regulations for the Narcotics Control Division of Cannabis under the Ministry of Public Health, Thailand, it was approved by the Cannabis Research Institute, Khon Kaen University, through grant project no. 65A103000130. Hemp extracts and residues were discarded according to the protocol.

The methodology for chitosan-microencapsulated hemp extract in this study was based on our previous study by Phupaboon et al. [14]. Briefly, dried powder of hemp leaf was extracted at a concentration of 10% (v/w) with distillated water using microwave extraction under optimal conditions to enhance the bioactive compounds and antioxidant capacity. Then, the composition (in 1000 mL) of the microencapsulated powder was prepared by including 500 mL of hemp extract juice mixed with 500 ml of 2% (w/v) chitosan media containing 2% (v/v) Tween 80 to retain their compounds through the spray-drying technique. The physical and morphological characterization of their microcapsules were observed by using field-emission scanning electron microscopy (FE-SEM) as shown in S1 Fig.

### Experimental design, dietary treatments, and chemical analysis

The study was designed as a completely randomized design (CRD) with five levels of mHLEs at 0, 2, 4, 6, and 8% of total DM substrate with 0.5 g of roughage to concentrate (R:C) ratio of 60:40. The details of the experimental run can be divided into three runs under triplicates of each treatment based on different parameters consisting of the gas production parameter (total 36 bottles), the nutrient degradability parameter (total 72 bottles, for 2 times assayed at 12 and 24 h), and the last parameter of VFA, NH_3_-N, along with the microbial population (total 108 bottles, for 3 times assayed at 12, 24 and 48 h). The concentrate was formulated with a combination of ingredients and minerals, as shown below in Table 1. Each of the dried materials was chemically analyzed for DM, ash, CP, and fibre fractions following the methodology of Thiex et al. [24]. The fibre contents (neutral detergent fibre; NDF and acid detergent fibre; ADF) were analyzed using the method of Van Soest et al. [25]. In addition, the mHLE was analyzed for phytonutrient values and antioxidative activities following the protocol described by Phupaboon et al. [26]. The phytonutrient values consisting of total polyphenolic content (TPC) was measured with Folin–Ciocalteu reagent absorbance at 765 nm, which expressed as mg gallic acid equivalents per gram of dry matter (mg GAE/g DM) and total flavonoid content (TFC) was determined based on colorimetric changes with 10% aluminum chloride solution read at 415 nm, which reported as mg quercetin equivalents per gram of dry matter (mg QUE/g DM). In addition, the antioxidative values in terms of 2,2-diphenyl-1-picrylhydrazyl (DPPH assay), 2,2′-azino-bis (3-ethylbenzothiazoline-6-sulfonic acid) (ABTS assay), radical scavenging inhibition, and ferric reducing antioxidant power (FRAP assay) were measured in the extract juice and microcapsule products as shown in Table 1.

**Table 1:** Chemical composition of feeds and mHLE supplement.

Items	Concentrate	Rice straw	mHLE
Ingredients (% as fed)			
Cassava chip	54.0		
Rice bran meal	17.0		
Palm kernel meal	13.0		
Soybean meal	10.5		
Urea	2.5		
Sulphur	1.0		
Salt	1.0		
Mineral mixed^1^	1.0		
Chemical composition			
Dry matter (DM, %)	90.5	89.4	92.6
	% dry matter (DM)		
Organic matter (OM)	92.2	85.4	93.9
Crude protein (CP)	14.6	2.4	21.5
Neutral-detergent fibre (NDF)	20.5	78.9	43.4
Acid-detergent fibre (ADF)	8.2	52.6	22.5
Phytonutrition content			
TPC (mg GAE/g DM)	-	-	240.0
TFC (mg QUE/g DM)	-	-	22.6
Antioxidative values			
DPPH inhibition (%)	-	-	69.9
ABTS inhibition (%)	-	-	53.0
FRAP capacity (mg TROE/g DM)	-	-	17.2

^1^ Mineral premix (contains per kg): vitamin A 10,000,000 IU; vitamin D 1,600,000 IU; vitamin E 70,000 IU; Fe 50 g; Mn 40 g; Zn 40 g; Cu 10 g; I 0.5 g; Se 0.1 g; Co 0.1 g; mHLE, microencapsulated of hemp leaf extract; TPC, total phenolic content; TFC, total flavonoid content; DPPH (2, 2-diphenyl-1-picrylhydrazyl) as DPPH radical scavenging activity; ABTS [2, 2’-azino-bis (3-ethylbenzothiazoline-6-sulfonic acid)] as ABTS radical scavenging activity; FRAP = ferric reducing antioxidant power; GAE, gallic acid equivalent; QUE, quercetin equivalent; TROE, Trolox equivalent.

### Rumen fluid and *in vitro* fermentation

Four rumen-fistulated dairy steers with an average live weight of 320 ± 10 kg were used as rumen fluid donors. The donor cattle in the experiment were provided *ad libitum* access to rice straw and received a concentrate diet at 0.5% of their body weight (BW) daily. The concentrate diet contained 145 g/kg crude protein (CP) and 810 g/kg total digestible nutrients (TDN), which was administered twice a day, at 6:00 AM and 4:00 PM. Approximately 1000 mL of total rumen fluid from four cows was collected using a suction pump and blended prior to the morning feeding. The sample was combined with the filtered rumen fluid and the prepared artificial saliva solution as ratio (1:2 mL/mL) and incubated at 39°C with continuous CO_2_ flushing [27]. Each mixed substrate plus treatment (total weight = 200 milligrams) was weighed and combined with 40 mL of rumen fluid medium into a sample bottle. The procedure for *in vitro* fermentation was based on the methodology of Blümmel and Ørskov [28] with some modifications. Gas production during incubation was measured at 0, 2, 4, 6, 8, 12, 24, 48, 72, and 96 h. Accordance with Ørskov and McDonald [29] model was fitted to the cumulative gas production of the data.

For the kinetic analysis during the fermentation of the ruminal fluid, the inoculum was directly measured following the inoculation at 12, 24 and 48 h for gas production and pH changes. For the other parameters, the rumen fluid samples were divided into two portions; the first portion of rumen fluid was centrifuged to collect the pellet and DNA extraction was used to identify the rumen microbiota through the real-time PCR technique. The second supernatant portion was provided with insoluble fibre by filtration followed by centrifugation and kept at -20°C for analysis of the ammonia nitrogen (NH_3_-N) content [30] and concentration of volatile fatty acids (VFAs) using GC instrument in accordance with Moss et al. [31]. The *in vitro* DM degradability (g/kg) after the incubation time at 12, 24 and 48 h were calculated according to the method of Van Soest et al. [25]. The concentrations of volatile fatty acids (VFAs) were chemically analyzed using a gas chromatograph (Nexis GC; Shimadzu Co., Kyoto, Japan). Additionally, the VFA proportions were used to estimate the rumen CH_4_ production, as follows; [CH_4_ production = 0.45 (acetate, mol/100 mol) – 0.275 (propionate, mol/100 mol) + 0.4 (butyrate, mol/100 mol)] [31].

### DNA extraction

The total gDNA template was prepared from 1.0 mL of the first portion of rumen fluid after fermentation times of 12, 24 and 48 h. The extraction using the QIAamp Fast DNA Stool Mini kit (Qiagen, Germany) was carried out in accordance with a procedure for pathogen detection that was outlined in the manufacturer’s instructions. The extracted DNA template was normalized by DI water and distilled water to measure the DNA concentration using DS-11 Spectrophotometers (DeNovix Inc., USA) and purified by the DNA standard by QIAquick Gel Extraction Kit (Qiagen, Germany) for consistent quantitative real-time PCR (qRT-PCR) analysis.

### Specific primers and quantitative real-time PCR analysis

The specific-species primers for investigation the dominant species in rumen microbiota through qRT-PCR used to amplify partial 16S rDNA in conserved V3-V4 regions were chosen from the previous research, namely *F*. *succinogenes*, *R*. *albus*, *R*. *flavefaciens* [32], *M*. *elsdenii* [33], *B*. *fibrisolvens* [34], *B*. *proteoclasticus* [35], and *Methanobacteriales* [36].

The qRT-PCR amplification was performed using the CFX Connect^TM^ Real-Time System (Bio-Rad, Singapore). The optimal temperature conditions used for annealing step were 55°C, and the reaction mixture was conducted in a final volume of 10 μL containing the following: Maxima SYBR Green qPCR Master Mix (Thermo Scientific™, USA) (5.0 μL), Fw and Rv primers (0.2 μL), purified DNA template (3 μL), and water (1.6 μL). The thermal cycling protocol was modified according to the procedure of Lee et al. [37]. The number of each species was counted in duplicate from each sample, and the mean value was calculated. The 10-fold serial dilutions of each standard DNA containing the target gene sequences of the relevant microbial group were used to create standard curves. The log10 gene copy number/mL of rumen fluid was used to express the absolute abundance of each microbial group or specific species [38].

### Statistical analysis

Data management and analysis were performed via a CRD platform using the PROC GLM procedure of SAS version 9.0 (SAS Inst. Inc., NC, USA). Differences between treatment means were determined by Tukey’s test, and differences among means with *P* < 0.05 and < 0.01 were accepted as showing statistically significant differences. Trends in mHLE-level responses were performed by orthogonal polynomials.

## Results

### Chemical composition of raw materials

Table 1 shows the results of the nutritive values of concentrate, rice straw, and mHLE supplementation as a percentage of DM, OM, CP, NDF and ADF. Subsequently, the phytochemical characteristics were measured by TPC, TFC, DPPH, ABTS radical scavenging inhibition, and FRAP-reducing power capacity in terms of phytonutrient values. The majority of phytonutrients found in mHLE had TPC (e.g., condensed tannins) more than TFC (e.g., saponins). In addition, the antioxidant capacity of mHLE found that the efficiency of DPPH, ABTS radical scavenging inhibition and FRAP-reducing power was greater than hemp meal extract.

### Nutrient degradation, and gas production

The *in vitro* dry matter degradability (IVDMD) at 12 and 24 h of post-fermentation when increasing the level of mHLE supplementation was quadratically increased ranging from 56.7 to 68.7% DM. Interestingly, the mHLE supplementation at 6% of total DM substrate was increasingly enhanced IVDMD during *in vitro* fermentation by 12, 24 and 48 h to 62.9, 68.7 and 72.7% DM, respectively (*P* < 0.05). In contrast, T_3_ supplementation did not enhance IVDMD at 48 h (*P* > 0.05). In addition, the gas production results are presented in Table 2, S2 Fig and S1 Table. Gas production kinetics consisting of the gas production rate constant for the insoluble fraction (c) was quadratically increased (*P* < 0.05) with mHLE supplementation at different level from 4 to 8% of total DM substrate. Whilst decreasing the gas production from the immediately soluble fraction (a), the gas production from the insoluble fraction (b), the potential extent of gas production (a + b), and cumulative gas (96 h) were statistically different (quadratic, linear and cubic effect; *P* < 0.05). These results conclude that the supplementation treatment in T_2_ to T_4_ levels of 4, 6 and 8% of total DM was significantly different (*P* < 0.05) when compared with treatment control (T_1_).

**Table 2:** Supplementation of mHLE on gas production kinetics and nutrient degradability.

Treatment	mHLE (%DM)	Gas kinetics^1^				Cumulative gas^2^ at 96 h	IVDMD (%DM)		
		a	b	c	a+b		12 h	24 h	48 h
1	0	-5.1^a^	122.3^a^	0.019^c^	111.2^a^	104.2^a^	56.7^b^	61.6^b^	67.6
2	4	-2.7^b^	97.7^b^	0.023^b^	97.3^b^	82.3^b^	62.7^a^	67.9^a^	72.4
3	6	-2.9^b^	99.5^b^	0.026^a^	96.6^b^	88.5^b^	62.9^a^	68.7^a^	72.7
4	8	-3.3^b^	88.3^b^	0.026^a^	85.0^b^	79.7^b^	62.0^a^	66.9^a^	69.6
SEM		0.48	4.28	0.01	4.41	3.49	0.75	0.67	1.20
Orthogonal polynomials									
Linear		0.06	0.05	0.15	0.04	0.60	0.13	0.24	0.74
Quadratic		<0.01	0.04	0.02	0.13	0.08	0.03	0.04	0.16
Cubic		0.36	0.82	0.07	0.91	0.03	0.22	0.15	0.93

^1^ Gas production kinetics, a, the gas production from the immediately soluble fraction (mL); b, the gas production from the insoluble fraction (mL); c, the gas production rate constant for the insoluble fraction (mL/h); a + b, the potential extent of gas production (mL).

^2^ Cumulative gas at 96 h (mL/0.2 g DM substrate).

^a-c^ Means with different superscripts within a column are significantly different (*P* < 0.05); treatments are expressed as mean and values are calculated from a minimum of three replicates; mHLE, microencapsulated of hemp leaf extract; IVDMD, in vitro dry matter degradability; SEM, standard error of mean.

### Rumen fermentation profiles

The ruminal pH and NH_3_-N concentration (at 12, 24 and 48 h) are shown in Table 3. The ruminal pH value ranged from 6.76 to 6.98 from all treatments. The result of ruminal pH at 24 h was quadratically impacted (*P* < 0.05) when increasing the level of mHLE supplementation. Interestingly, the pH value is correlated with the efficiency of nutrient degradability (IVDMD) due to the enhancement in the ruminal microbial population, which might effectively degrade feed activity. Furthermore, the mHLE supplementation of each treatment at different time points was quadratically and linearly increased (*P* ≤ 0.01, *P* = 0.05) the ruminal NH_3_-N concentration at 13.4, 13.6 and 19.5 mg/dL when compared with the control experiment (T_1_) at 10.5, 11.3 and 13.9 mg/dL, respectively. Especially, the supplementation of 6% of total DM substrate was significantly increased (*P* < 0.05) from 13.4 to 19.5 mg/dL for the observation time at 12 to 48 h. Furthermore, the rumen NH_3_-N concentration of the current result was markedly increased (*P* < 0.05) under the efficiency of supplemented mHLE-based bioactive compounds formulated by the gelation action of chitosan. These results can be used to conclude that an increase in NH_3_-N concentration is caused by increased microorganism protein synthesis, thus directly resulting in a higher rumen by-pass (Table 3).

**Table 3:** Supplementation of mHLE on *in vitro* ruminal pH and ammonia-nitrogen concentration.

Treatment	mHLE (%DM)	pH			Ammonia nitrogen (mg/dL)		
		12 h	24 h	48 h	12 h	24 h	48 h
1	0	6.94	6.91^c^	6.95	10.5^b^	11.3^b^	13.9^c^
2	4	6.95	6.96^a^	6.86	12.8^a^	12.5^a^	15.7^b^
3	6	6.95	6.98^a^	6.76	13.4^a^	13.6^a^	19.5^a^
4	8	6.92	6.93^b^	6.83	9.5^c^	10.3^b^	18.6^a^
SEM		0.02	0.01	0.05	0.28	0.31	0.44
Orthogonal polynomials							
Linear		0.12	0.08	0.35	0.53	0.61	<0.01
Quadratic		0.26	0.02	0.24	0.01	0.05	0.94
Cubic		0.57	0.67	0.87	0.35	0.04	0.62

^a-c^ Means with different superscripts within a column are significantly different (*P* < 0.05); treatments are expressed as mean and values are calculated from a minimum of three replicates; mHLE, microencapsulated of hemp leaf extract; SEM, standard error of mean.

The results of total VFA, individual VFA proportions and methane production are shown in Table 4. The supplementation of mHLE (4 to 8% of total DM substrate) had significantly quadratic effect (*P* < 0.05) the proportion of propionate (C_3_) and butyrate. It also resulted in the highest concentrations of propionate (C_3_) and total VFA compared to the control group in the experiment. On the other hand, when the supplementation level was increased, the proportion of acetate (C_2_) and acetate to propionate (C_2_:C_3_) ratios were subsequently decreased, additionally, the effectiveness of mHLE supplement (6% total DM substrate) was quadratically decreased (*P* < 0.05) in the butyrate content. Furthermore, the calculated CH_4_ production after 12, 24 and 48 h of post-fermentation was linearly decreased when the mHLE supplement level was increased. Importantly, the 6% total DM substrate of mHLE supplement resulted in lower CH_4_ proportion (%) than those in other treatments and control group. This result found a significant reduction in methane (CH_4_) production by 8.9%, 11.3%, and 16.6%, respectively.

**Table 4:** Supplementation of mHLE on *in vitro* volatile fatty acids (VFA), total VFA production, and methane (CH_4_) production.

Treatment	mHLE (%DM)	VFA (mol/100 mL)			C_2_:C_3_	Total VFA (mmol/L)	Methane production (%)		
		C_2_	C_3_	C_4_			12 h	24 h	48 h
1	0	70.2	22.5^b^	7.3^b^	3.1	69.5	26.9^a^	30.2^a^	34.3^a^
2	4	66.3	25.0^a^	8.7^a^	2.7	76.2	26.0^b^	29.3^b^	33.4^b^
3	6	60.3	28.5^a^	7.2^b^	2.1	86.9	24.5^c^	26.8^c^	28.6^c^
4	8	67.6	24.4^a^	8.0^a^	2.8	73.8	25.3^d^	28.0^d^	32.7^c^
SEM		1.40	0.52	0.39	0.16	2.48	0.06	0.04	0.04
Orthogonal polynomials									
Linear		0.87	0.94	0.67	0.68	0.24	0.01	<0.01	0.02
Quadratic		0.30	0.04	0.04	0.25	0.65	0.58	0.35	0.49
Cubic		0.29	0.46	0.12	0.41	0.57	0.47	0.29	0.37

^a-d^ Means with different superscripts within a column are significantly different (*P* < 0.05); treatments are expressed as mean and values are calculated from a minimum of three replicates; mHLE, microencapsulated of hemp leaf extract; VFA, volatile fatty acids; C_2_, acetate; C_3_, propionate; C_4_, butyrate; C_2_:C_3_, acetate to propionate ratio; SEM, standard error of mean.

### Rumen microorganisms

Seven specific species, namely *Fibrobacter succinogenes*, *Ruminococcus albus*, *Ruminococcus flavefaciens*, *Megasphaera elsdenii*, *Butyrivibrio fibrisolvens* and *Butyrivibrio proteoclasticus* were significantly changed (linear, quadratic and cubic effect; *P* ≤ 0.05) by the effectiveness of mHLE supplementation at different several time of the *in vitro* fermentation, as shown in Table 5. Interestingly, the present results showed that the mHLE supplementation (4 to 8% total DM substrate) were decreased dominant species in the phyla of fibrolytic and cellulolytic bacteria: *F*. *succinogenes* (*P* < 0.05), *R*. *albus* (*P* < 0.05) and *R*. *flavefaciens* (*P* < 0.05), along with Acidobacteria: *M*. *elsdenii* (*P* < 0.05), as well as Biohydrogenation bacteria: *B*. *fibrisolvens* (*P* ≤ 0.05) and *B*. *proteoclasticus* (*P* < 0.05). Mostly, the mHLE supplement (at 4 to 6% total DM substrate) had linearly increased the population of *R*. *albus* (10^8^ copies/mL) as a highest dominant species. Another relative abundance of *R*. *flavefaciens*, *M*. *elsdenii* and *F*. *succinogenes* (10^7^ copies/ml) were increased (*P* < 0.05) in 6% mHLE compared with control. Moreover, the result of the present work found that the population of protozoa and methanogens, specifically *Methanobacterials* (10^7^ copies/mL) was significantly reduced (linear and quadratic effect; *P* < 0.05) when fed the mHLE (Table 5).

**Table 5:** Supplementation of mHLE on rumen microbial population.

Species	Incubation time	mHLE (%DM)				SEM	Orthogonal polynomials		
		0	4	6	8		L	Q	C
*F*. *succinogenes* (×10^7^ copies/mL)	12 h	0.5	0.4	0.4	0.6	0.81	0.70	0.49	0.97
	24 h	3.1^a^	1.2^b^	0.5^b^	0.2^b^	1.12	0.01	0.18	0.66
	48 h	0.8	0.8	0.5	0.5	1.35	0.69	0.96	0.44
*R*. *albus* (×10^8^ copies/mL)	12 h	0.7	0.6	0.5	0.3	0.85	0.25	0.91	0.37
	24 h	1.6^a^	1.4^b^	0.6^b^	0.3^c^	0.24	0.01	0.91	0.46
	48 h	2.9	1.9	1.3	0.9	1.95	0.06	0.68	0.90
*R*. *flavefaciens* (×10^7^ copies/mL)	12 h	1.1^a^	0.9^b^	0.7^b^	0.5^c^	0.63	0.02	0.90	0.84
	24 h	2.3^a^	0.8^b^	0.8^b^	0.4^b^	1.69	0.02	0.22	0.30
	48 h	1.9^a^	0.5^b^	0.9^b^	0.7^b^	0.82	0.01	0.02	0.03
*M*. *elsdenii* (×10^7^ copies/mL)	12 h	0.9	1.4	1.0	1.0	0.54	0.90	0.10	0.06
	24 h	1.5^a^	1.4^a^	1.0^b^	0.9^c^	0.55	0.01	0.96	0.45
	48 h	0.9	0.6	0.7	1.1	0.89	0.45	0.09	0.83
*B*. *fibrisolvens* (×10^7^ copies/mL)	12 h	1.2	1.8	1.4	0.9	1.04	0.19	0.05	0.41
	24 h	2.4	1.8	3.1	1.9	1.72	0.98	0.45	0.05
	48 h	2.4	1.4	3.0	2.9	2.11	0.35	0.49	0.23
*B*. *proteoclasticus* (×10^7^ copies/mL)	12 h	1.5^c^	1.9^b^	2.4^a^	2.1^b^	0.94	0.04	0.15	0.51
	24 h	2.1	2.2	2.3	2.0	0.73	0.80	0.28	0.60
	48 h	1.5	1.4	1.4	1.7	1.53	0.69	0.66	0.83
*Methanobacteriales* (×10^7^ copies/mL)	12 h	2.5	1.8	1.4	1.4	1.42	0.04	0.53	0.37
	24 h	1.3	1.1	1.1	1.1	0.59	0.99	0.03	0.33
	48 h	0.9	0.8	0.8	0.6	0.50	0.17	0.65	0.62

^a-c^ Means with different superscripts within a column are significantly different (*P* < 0.05); treatments are expressed as mean and values are calculated from a minimum of three replicates; mHLE, microencapsulated of hemp leaf extract; SEM, standard error of mean; L, linear; Q, quadratic; C, cubic.

## Discussion

### Composition of raw materials and mHLE supplementations

The chemical compositions of hemp meal and mHLE supplements have been reported to contain high concentrations of phytonutrients, in particular TPC, TFC and antioxidant capacity. The results were similar to the findings of Kleinhenz et al. [39], who reported the phytochemical composition of hemp flower, leaf and chaff extracts. These samples were reported to have a high chemical composition in the ranges of percentages of CP (13.0–24.5), NDF (27.9–44.7), ADF (18.0–20.8), ether extract (EE; 3.2–8.9), non-structural carbohydrate (NCS; 4.7–6.3) and ash (EE; 21.2–25) in the unit of DM. Numerous studies have reported the biological activities of hemp plant extract in terms of phenolic compounds (PC) such as flavonoids, stilbenoids, and lignans, in particular the phenol amide *N*-trans-caffeoyltyramine and the lignanamides cannabisins A and B, which have shown a potent inhibitory effect on the oxidation of DPPH and ABTS as well as the reduction of ferric [40–43].

### *In vitro* gas production kinetics and nutrient degradability

Cumulative gas production and gas production kinetics are correlated with nutrient degradability obtained from this research. The mHLE supplement at 4 and 6% of total DM substrate impacted the immediately soluble fraction (a) and insoluble fraction (b). These findings are consistent with previous study, suggesting that it could potentially reduce rumen gas production by trapping electrons and diverting them towards the formation of methane or ammonia, influenced by interactions among rumen microbial consortia within biofilms [44]. Similarly, previous studies by Mohammadabadi [45] indicated that a diet supplemented with 10% hemp seeds reduced gas formation. However, hemp seeds had the highest digestibility by camel cellulolytic bacteria after 24, 48 and 72 h of incubation. According to Jensen et al. [46] studied the effects of THC and/or flavonoid glycosides from industrial hemp (*Cannabis sativa* L.) extract incubated with a standard feed and noted significantly reduced methane production compared with controls, and they exhibited high influences on feed degradability and volatile fatty acid patterns.

### Ruminal pH and ammonia-nitrogen (NH_3_-N) concentration

The ruminal pH fermentation at 12 to 48 h was not changed by either the R:C ratio or the mHLE supplement, with an average of 6.7 to 6.9. Accordance with previous studies, a ruminal pH of neutral (6.6–7.3) covered the slightly acidic (6.1–6.5) condition in feed with a high concentration of roughage to maintain the growth rate of dominant species, in particular cellulolytic bacteria [47, 48]. Additionally, Thao et al. [17] showed a ruminal NH_3_-N profile ranging from 15.8–30.0 mg/dL after incubation with the supplementation of chitosan extract and shrimp shell meal with an R:C ratio of both 60:40 and 40:60. Another important finding was that increasing dragon fruit peel pellet (DFPP) supplementation as a phytonutrient substate increased mean NH_3_-N concentration from 14.3 to 20 mg/dL, which is suitable for nutritional degradability and microbial protein synthesis. In contrast, Phesatcha et al. [49] and Wanapat et al. [50], reported that *Mitragyna speiosa* leaf supplementation (MSLP) and mangosteen peel powder (MPP) used as phytonutrient compounds resulted in lower ruminal NH_3_-N concentrations, probably due to condensed tannins and saponins in dry matter that safeguarded the protein degradation and generated the protein-CT binding complex, which in the case of inhibitors for decreasing the protein degradability and the rumen microbiota hampered by NH_3_-N synthesis.

### Volatile fatty acid concentration and methane production

The current result of total VFA production is not affected in particular the mHLE supplemented in T_3_ at 6% of total DM substrate was increased when compared with T_1_, T_2_, and T_4_; this was similar to the finding of Jensen et al. [46], who reported that the addition of hemp extract type *Finola* F25 on maize silage feed had the highest total VFA concentration at 120 mol/L. According to Wang et al. [12], who reported the effect of hemp oilseeds combined with coconut oil on rumen fermentation at dosages of 70 g/kg DM decreased and CH_4_ emissions increased up to 16%. Additionally, Wanapat et al. [50], calculated the CH_4_ production was lower in treatments with a mixture of herb plants including lemongrass, peppermint and garlic (LPG) powder supplements compared to controls, and it tended to exhibit the lowest value in the LPG at 27.9 mL/100 mL. However, in accordance with Goiri et al. [19], the decreased rumen CH_4_ synthesis caused by the supplementation of chitosan extract could be related to the enhancement of C_3_ formation, which required the metabolism of hydrogen, resulting in decreased methanogenesis. The current concept aligns with additional research findings on feed supplemented with various agro-nutrients, specifically condensed tannins, saponins, and/or bioactive compounds, which inhibit the microbiota in ruminant CH_4_ mitigation. This is done to study essential oils such as hemp seed, sunflower, or tobacco cake [51], as well as some fractions of insect protein [52, 53].

### Rumen microorganisms

Consequently, the polyphenolic, flavonoids, and antioxidative components found in mHLE and their compounds have the potential to influence *Methanobacteriales* (10^7^ copies/mL), while cellulolytic bacteria demonstrated a higher population of *R*. *albus* (10^8^ copies/mL) compared to supplemented mHLE at 4 to 8% of total DM substrate. This finding agrees with Ultee et al. [54] explained the idea of cellulolytic bacteria losing chemiosmotic control by plant phytonutrient-based bioactive activity based on (i) bioactive compound activity alters protein translocation; (ii) phosphorylation processes; (iii) ion gradients transport and (iv) other enzyme-dependent processes. In contrast to earlier findings of Koike et al. [32], who reported three cellulolytic microorganisms, *F*. *succinogenes* was the most dominant species of digesta and rumen fluid of swamp buffalo (*Bubalus bubalis*) in a range of 10^6−9^ copies/mL, shadowed by *R*. *albus* and *R*. *flavefaciens* at 10^4−6^ and 10^5−6^ copies/mL, respectively. In addition, the effect of mHLE supplementation containing TPC, TFC and antioxidant capacity was conducted in this study, when increasing the concentration of their supplementation in the *in vitro* rumen fermentation, it continuously decreased the population of protozoa and methanogens, particularly *Archaeoglobus fulgidus* and *Methanobacteriales* (e.g., *Methanobacterium formicicum*). Similarly, Phesatcha et al. [49] estimated the efficacy of *Mitragyna speciosa* leaf supplemented into diet in the *in vitro* fermentation, it reduced the population of protozoa and methanogens. It could conceivably be hypothesized that some decrease in the protozoa population may affect the methanogen population and methanogenesis because methanogens have symbiotic interactions with protozoa and they offer hydrogen as a substrate for CH_4_ production [55, 56]. Furthermore, the bioactive compounds exhibit prompt effects on rumen methanogens by their interaction with proteinaceous adhesion. This interaction leads to the suppression of methanogen growth, reduction in interspecies hydrogen transfer, and prevents the development of the methanogen-protozoa complex [57].

## Conclusions

This research has demonstrated that supplementation of mHLE, formulated with chitosan as a wall material, effectively retains phytonutrients and antioxidative compounds from hemp extract using the spray-drying technique. The supplementation of mHLE at 6% of total DM substrate increasingly resulted in improving the *in vitro* nutrient degradability, ruminal fermentation end-products, especially propionate production, and decreased methanogens and methane emission. Therefore, it is plausible that the mHLE supplement demonstrates efficacy as a dietary-based phytonutrient component and may have prospective use as a feed additive.

## Supporting information

S1 Fig SEM micrographs of surface morphology and microstructure of spray dried chitosan microencapsulated of hemp leaves extract (mHLE). (PDF)

S2 Fig Effect of mHLE on cumulative gas produced curves by during in an *in vitro* fermentation at 0 to 96 h of incubation times. The treatment (T1-T4) was added with mHLE at 0, 2, 4, 6, and 8% of total DM substrate.
(PDF)

S1 Table Total amount of gas produced by mHLE during in an *in vitro* fermentation at o to 96 h of incubation. (XLSX)
