# Supplementary material for: Bioefficiency of microencapsulated hemp leaf phytonutrient-based extracts to enhance in vitro rumen fermentation and mitigate methane production
Source: PLoS One. 2024 Oct 31;19(10):e0312575. doi: 10.1371/journal.pone.0312575 (PMC11527300; doi:10.1371/journal.pone.0312575)
Supplement: S1 Fig — (PDF) [file pone.0312575.s001.pdf]

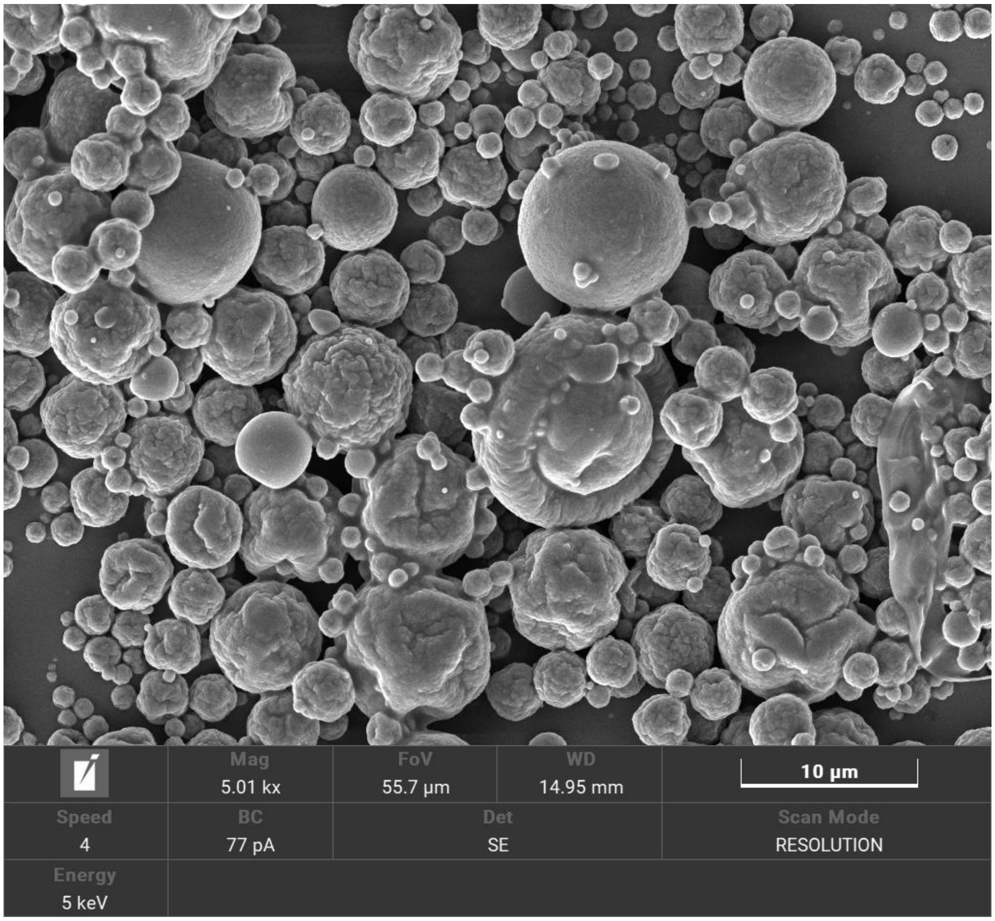


**S1 Fig.** SEM micrographs of surface morphology and microstructure of spray dried chitosan microencapsulated of hemp leaves extract (mHLE).
