# Supplementary material for: Bioefficiency of microencapsulated hemp leaf phytonutrient-based extracts to enhance in vitro rumen fermentation and mitigate methane production
Source: PLoS One. 2024 Oct 31;19(10):e0312575. doi: 10.1371/journal.pone.0312575 (PMC11527300; doi:10.1371/journal.pone.0312575)
Supplement: S2 Fig — The treatment (T1-T4) was added with mHLE at 0, 2, 4, 6, and 8% of total DM substrate. (PDF) [file pone.0312575.s002.pdf]

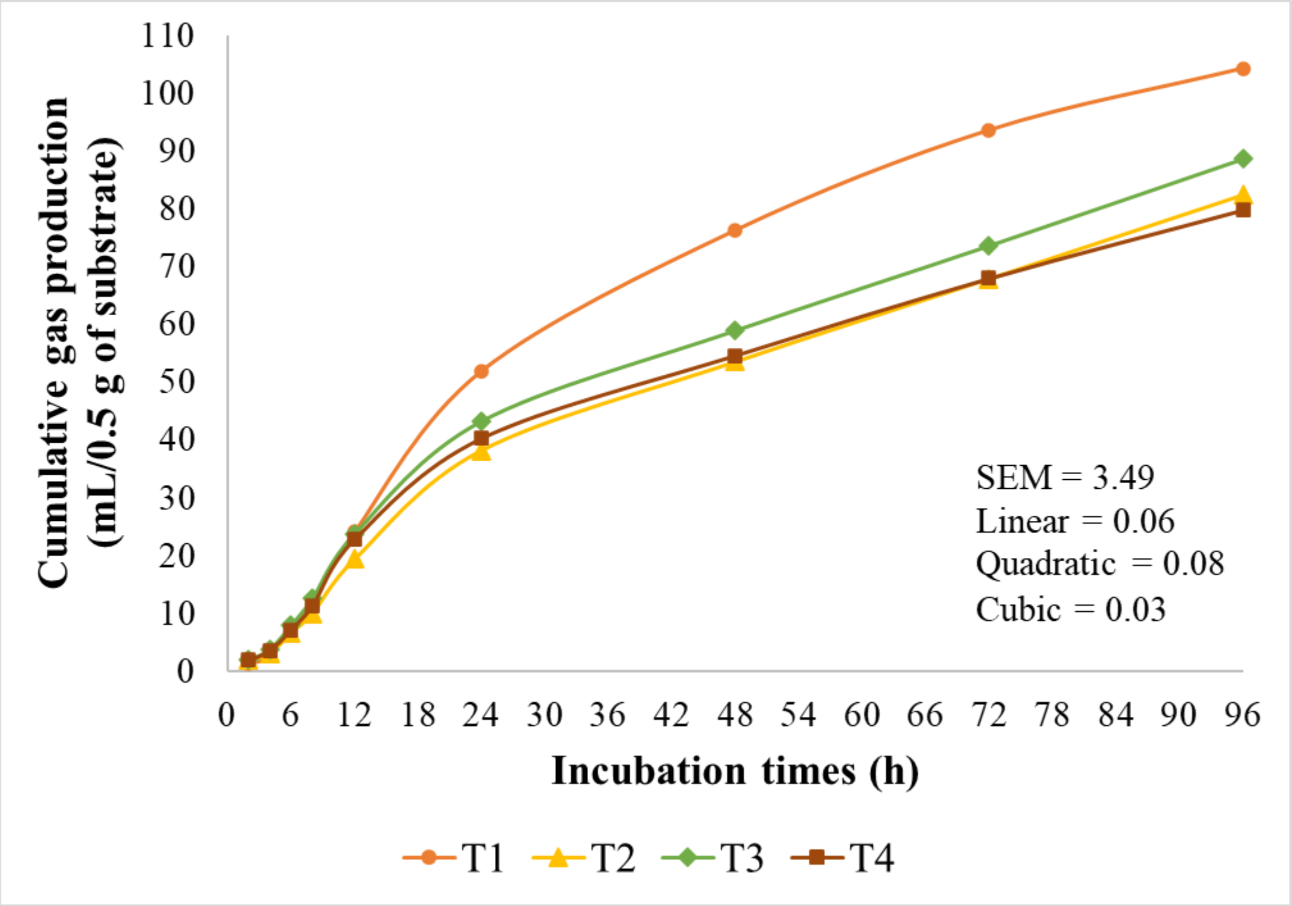

S2 Fig. Effect of mHLE on cumulative gas produced curves by during in an *in vitro* fermentation at 0 to 96 h of incubation times. The treatment (T1-T4) was added with mHLE at 0, 2, 4, 6, and 8% of total DM substrate.
